# ASPP2 attenuates triglycerides to protect against hepatocyte injury by reducing autophagy in a cell and mouse model of non-alcoholic fatty liver disease

**DOI:** 10.1111/jcmm.12364

**Published:** 2014-09-25

**Authors:** Fang Xie, Lin Jia, Minghua Lin, Ying Shi, Jiming Yin, Yin Liu, Dexi Chen, Qinghua Meng

**Affiliations:** a Beijing You An Hospital, Affiliated Hospital of Capital Medical UniversityBeijing, China; b Beijing Institute of HepatologyBeijing, China

**Keywords:** ASPP2, NAFLD, triglyceride, autophagy

## Abstract

ASPP2 is a pro-apoptotic member of the p53 binding protein family. ASPP2 has been shown to inhibit autophagy, which maintains energy balance in nutritional deprivation. We attempted to identify the role of ASPP2 in the pathogenesis of non-alcoholic fatty liver disease (NAFLD). In a NAFLD cell model, control treated and untreated HepG2 cells were pre-incubated with GFP-adenovirus (GFP-ad) for 12 hrs and then treated with oleic acid (OA) for 24 hrs. In the experimental groups, the HepG2 cells were pre-treated with ASPP2-adenovirus (ASPP2-ad) or ASPP2-siRNA for 12 hrs and then treated with OA for 24 hrs. BALB/c mice fed a methionine- and choline-deficient (MCD) diet were used to generate a mouse model of NAFLD. The mice with fatty livers in the control group were pre-treated with injections of GFP-ad for 10 days. In the experimental group, the mice that had been pre-treated with ASPP2-ad were fed an MCD diet for 10 days. ASPP2-ad or GFP-ad was administered once every 5 days. Liver tissue from fatty liver patients and healthy controls were used to analyse the role of ASPP2. Autophagy, apoptosis markers and lipid metabolism mediators, were assessed with confocal fluorescence microscopy, immunohistochemistry, western blot and biochemical assays. ASPP2 overexpression decreased the triglyceride content and inhibited autophagy and apoptosis in the HepG2 cells. ASPP2-ad administration suppressed the MCD diet-induced autophagy, steatosis and apoptosis and decreased the previously elevated alanine aminotransferase levels. In conclusion, ASPP2 may participate in the lipid metabolism of non-alcoholic steatohepatitis and attenuate liver failure.

## Introduction

Non-alcoholic fatty liver disease (NAFLD) is a condition that varies in severity from lipid accumulation to steatohepatitis and even cirrhosis. NAFLD is associated with insulin resistance and obesity [1,2] and induces a wide spectrum of liver damage [3]. At present, NAFLD occurs in one-third of the adult population and is increasingly observed in children in developed countries [4]. Although the pathogenesis of NAFLD is unclear, NAFLD has been linked to lipid accumulation and lipid metabolism dysfunction [5,6].

Lipid metabolism is critical in the progression of NAFLD, and autophagy plays an important role in lipid metabolism. In addition to its role in the elimination of intracellular pathogens and the removal of damaged organelles [7], autophagy has multiple pathophysiological and physiological functions in NAFLD. Autophagy can be induced by free fatty acids *in vitro* [8], and insulin resistance has been associated with autophagy impairment in NAFLD [9,10]. However, autophagy appears to play a paradoxical role in cell survival and death. In chemotherapy, the autophagic response plays a protective role in impeding eventual cell death [11–13]. However, recent studies have indicated that when autophagy was inhibited, there was decreased apoptosis [14,15].

Significant evidence has demonstrated that p53 plays a major role in the pathogenesis of NAFLD [16,17]. Zoltan *et al*. reported that p53 plays a central role in NAFLD, and the partial inhibition of p53 activity markedly diminished hepatic steatosis in high-fat-diet-fed mice [18]. Tomit reported that p53 knockout mice fed a methionine- and choline-deficient (MCD) diet for 8 weeks developed less steatosis compared with their wild-type littermates [17].

P53-2 (ASPP2) is a pro-apoptotic regulator that enhances apoptosis by binding to p53 [19,20]. ASPP2 is involved in many biological pathways [21] and has been implicated in the pathogenesis of NAFLD. This study aimed to elucidate whether ASPP2 can decrease triglyceride (TG) accumulation and to clarify the relationship between ASPP2, apoptosis and autophagy in NAFLD. Our results suggest that the overexpression of ASPP2 could decrease the accumulation of TGs and inhibit autophagy and apoptosis.

## Materials and methods

### Cell culture and treatment

The human hepatoblastoma cell line HepG2 was grown in DMEM containing 10% foetal bovine serum, penicillin (100 U/ml), and streptomycin (100 μg/ml). The HepG2 cells were divided into four groups. The control groups consisted of HepG2 cells treated with or without 400 μM oleic acid (OA) for 24 hrs; these cells were pre-incubated with GFP-adenovirus (GFP-ad) for 12 hrs. In the experimental groups, the HepG2 cells were pre-incubated with ASPP2 adenovirus (ASPP2-ad) or ASPP2 siRNA for 12 hrs and then treated with 400 μM OA for 24 hrs. FuGENE HD transfection reagent (Promega, Madison, WI, USA) was used for the transfection of ASPP2 siRNA.

### Lipid extraction

The cells were collected and washed with PBS. Small interfering RNAs (siRNA) specific to ASPP2 (target sequence: 5-AAGTTGCTGAGCAGGAGAAAC-3) and a nonspecific control were synthesized by Genepharmer. ASPP2 siRNA was transfected into HepG2 cells by using the FuGENE HD transfection reagent (Promega). The TG mass in the cells was determined by using a commercial TG determination kit (Applygen Technologies Inc., Beijing, China).

### Immunofluorescence

The cells were fixed with 10% paraformaldehyde/PBS, incubated with 1% Triton X-100/PBS for 10 min., and blocked with 3% BSA/PBS; then, a mouse anti-M30 antibody (produced by our laboratory) was used to detect early apoptosis. Nuclei were stained with 4,6-diamidino-2- phenylindole. M30 immunoreactivity was detected by using a fluorescence microscope (Nikon Eclipse 80iNikon Eclipse 80i,Shinagawa-ku, Tokyo, Japan). More than 1000 cells were counted to quantitate apoptosis in each examination.

### Lactate dehydrogenase assay

The level of lactate dehydrogenase (LDH) in the cells was determined by using a LDH assay kit (Applygen Technologies Inc, Beijing, China).

### Western blot analysis

The cells were pelleted and lysed in lysis buffer (10 mM HEPES pH 7.4, 1 mM EDTA, 0.15 M NaCl, 1% Triton X-100, 1 mM EGTA, 0.5% NP-40, and 100 mg/ml PMSF) for 30 min. The cell lysates were centrifuged at 8000 g at 4°C. The proteins were detected by using SDS-PAGE and transferred to nitrocellulose membranes. The membranes were first blocked with 6% non-fat milk and then incubated with the appropriate primary antibodies (ASPP2, LC3-II; from St. Louis, USA) at 4°C overnight. The membranes were washed three times with PBS and then incubated with the appropriate secondary antibodies for 1 hr at room temperature. X-ray film was used to record the protein bands.

### Electron microscopy

The treated and untreated HepG2 cells and liver tissue samples were collected and fixed with 3% glutaraldehyde in 0.2 M sodium cacodylate, pH 7.4; they were then washed three times with 1× PBS. After dehydration by using an ethanol series, the cells were embedded in Epon. Images were acquired with a transmission electron microscope.

### Oil Red O staining and the determination of the intracellular lipid accumulation

The HepG2 cells were incubated for 24 hrs with 400 μM OA. The cells were cultured on glass cover slips and stained with Oil Red O (5 mg/ml; St. Louis, USA) for 20 min. at 37°C to visualize the intracellular lipid droplets. Images were acquired with a Nikon Eclipse 80iNikon Eclipse 80i,Shinagawa-ku, Tokyo, Japan laser scanning confocal microscope. The intracellular TG content was quantified by using a commercial kit (PPLYGEN, Beijing, China).

### Mice and diets

Male BALB/c mice (Academy of Military Medical Sciences, China) were randomly assigned to the experimental group (MCD) or the control group (MCS). The mice in the MCD and MCS groups were fed an MCD Diet and MCS diet [the same diet as MCD with the addition of sufficient dl-methionine (3 g/kg) and choline bitartrate (2 g/kg)], respectively. The diets were purchased from Nantong (China). All studies were approved by the Ethical Committee of the You An Hospital Center, affiliated Hospital of Capital Medical University, Beijing, and all animal care complied with the applicable health guidelines. ASPP2+/− mice (BALB/c) will be used in our future experiments (and to ensure the accuracy and continuity of this experiment). We developed an NAFLD model in BALB/c mice. After 10, 20 and 30 days of MCD/MCS diet administration, 5–8 rats from each group were anaesthetized and killed, and their livers were removed. Ultrasonic histopathological analysis and serum alanine aminotransferase (ALT), aspartate aminotransferase (AST), TG and cholesterol (CHOL) activity assays were used to verify the development of steatosis and liver injury. We further studied the effect of ASPP2 on the lipid profile. The GFP-ad group mice were fed a MCD diet pre-treated with GFP-ad (60,000 viral particles/mouse) as the control. The ASPP2-ad group was fed a MCD diet pre-treated with ASPP2-ad (60,000 viral particles/mice) as the experimental group. Ten days later, the autophagy/apoptosis markers and lipid metabolism mediators were assessed.

### Liver tests

The serum ALT, AST, TG and CHOL levels were quantitated with an AU400 automatic biochemical analyzer (Shinjuku, Tokyo, Japan, Japan).

### Patients and immunofluorescence

Moderate NAFLD patients were recruited from You An hospital, Beijing. As approved by the Ethics Committee of You An Hospital, Beijing, normal liver tissues were obtained from liver transplant donors as the healthy control group; informed consent was obtained from all patients. Immunofluorescence was observed as previously described.

### Data analysis

All data are expressed as the mean ± SEM and represent at least three independent experiments. Statistical significance between groups was determined by using Student's *t*-test. Differences were considered statistically significant at confidence levels of *P* < 0.05 (*) or *P* < 0.01 (**).

## Results

### ASPP2 overexpression reduced the level of TG and autophagy in OA-treated HepG2 cells

**Fig. 1:**
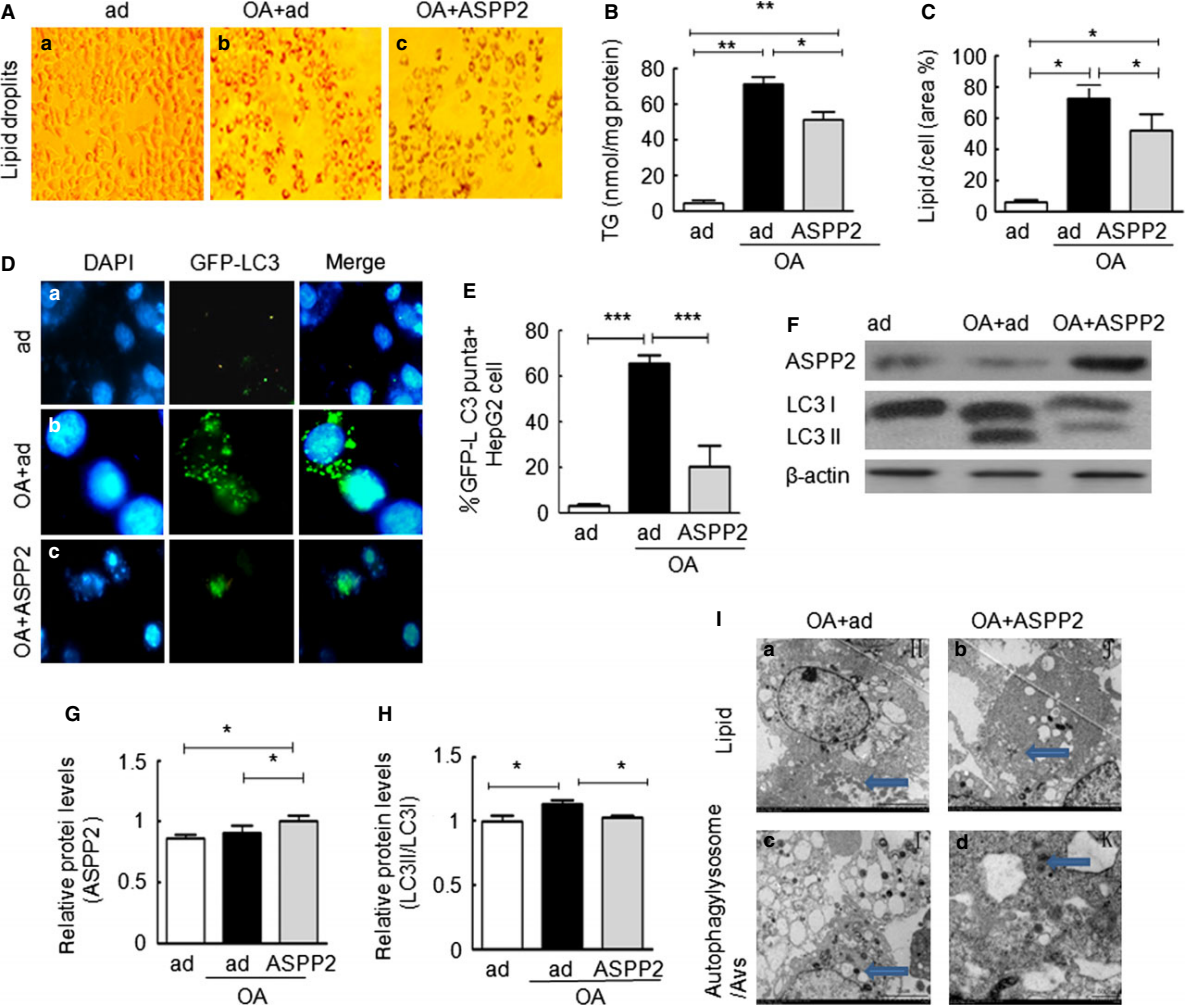
ASPP2 overexpression affects the induction of lipid accumulation and autophagy. HepG2 cells were treated with 400 μM OA for 24 hrs in the presence or absence of ASPP2-ad overexpression. (**A**–**C**) Oil Red O staining; TG content and lipid areas in the HepG2 cells exhibited markedly diminished steatosis in the presence of ASPP2-ad overexpression. (**D** and **E**) Detection of autophagy by using plasmids encoding GFP-LC3 and immunofluorescence. Representative images are shown (400×). (**F**) Western blotting analysis of the ASPP2 and LC3 I/II protein levels. (**G** and **H**) The relative protein levels of ASPP2 and LC3II/LC3I were normalized with β-actin. (**I**) Electron micrographs of HepG2 cells showing lipids, autophagylysosmes and autophagic vacuoles (AVs) after OA treatment with or without ASPP2-ad. (**I-a** and **I-b**) The arrows indicate lipids. (**I-c**) The arrow indicates an autophagic lysosome. (**I-d**) The arrow indicates an AV; *P < 0.05; **P < 0.01.

To investigate the role of ASPP2 in the process and mechanism of NAFLD, we constructed a NAFLD model by using HepG2 cells as previously described [1]. The results showed that the OA- and GFP-ad (ad)-treated group significantly induced TG accumulation compared with the Ad treatment alone (Fig. 1A-a and -b). Significantly fewer lipid droplets were observed in the ASPP2-ad treated group (Fig. 1A-c). The intracellular TG and lipid levels were further quantified as shown in Figure 1B and C. These levels confirmed that the accumulation of lipids and TGs induced by OA treatment was significantly decreased by ASPP2 overexpression. Recent studies have shown that autophagy plays a critical role in NAFLD and that ASPP2 inhibits autophagy [22]; therefore, we examined whether the elimination of excessive intracellular lipids was associated with ASPP2-mediated autophagy. The level of autophagy was analysed *via* the expression of microtubule-associated protein light chain 3 (LC3). In this study, we identified the level of autophagy based on the number of GFP-LC3 puncta and the LC3-I/LC3-II ratio, which are both markers of autophagy. The percentages of HepG2 cells that contained GFP-LC3 puncta were determined in eight non-overlapping fields. Each experiment was repeated three times. Immunofluorescence and western blot (WB) analyses showed that the levels of autophagy in the OA plus Ad-treated group were higher than those in the Ad-treated control group (Fig. 1D-a, -b and F-lanes 1 and 2 and H). However, the levels of autophagy observed in the OA plus ASPP2-ad group were lower compared with the OA plus Ad-treated group (Fig. 1D-b and -c, F-lanes 2 and 3, and H). The OA- plus ASPP2-ad-treated group had increased expression of ASPP2 compared with the OA- plus GFP-ad-treated group (Fig. 1G). Electron microscopy (EM) was useful for observing the autophagy and lipid contents of the cells. To further confirm that the ASPP2 decreased the levels of lipids and autophagy, we performed an EM assay. The experiment revealed a large number of autophagic lysosomes and lipids in the OA-treated group, with only a small number of autophagic vacuoles and low lipid levels in the ASPP2-ad group (Fig. 1I). This result supports the findings in Figure 1A–G, which indicate that ASPP2 overexpression can significantly reduce the level of autophagy, as well as the lipid accumulation in OA-treated HepG2 cells.

### ASPP2 overexpression can significantly reduce the apoptosis of OA-treated HepG2 cells

**Fig. 2:**
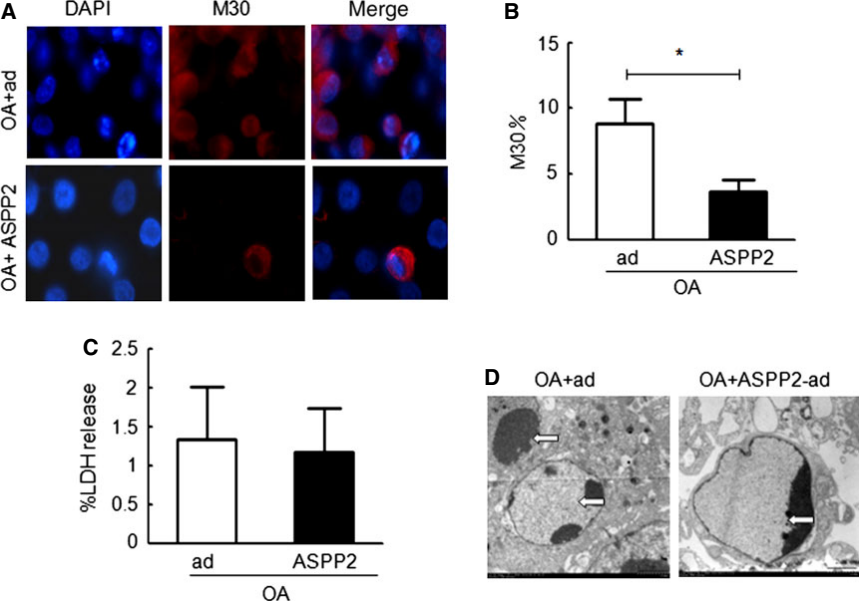
ASPP2 inhibits apoptosis in HepG2 cells. HepG2 cells were treated with 400 μM of OA for 24 hrs in the presence or absence of ASPP2-ad overexpression. (**A**) The detection of apoptosis by using an anti-M30 antibody. Original magnification (400×). (**B**) Early apoptotic cells. (**C**) The levels of LDH released by the cells. The data (mean ± SEM) represent three independent experiments. (**D**) Electron micrographs of HepG2 cells showing apoptotic cells after OA treatment with or without ASPP2-ad. The arrows indicate apoptotic cells; *P < 0.05; **P < 0.01.

Apoptosis, which promotes inflammation and interferes with hepatocyte function, plays an important role in the progression of NAFLD [23]. ASPP2 selectively stimulates the p53 (and p73/p63) transactivation of target genes, in addition to mediating p53-independent functions and inhibiting cell growth. Importantly, the ASPP2 allele targeted in mouse models confirms that ASPP2 is a tumour suppressor [24]. In this study, we aimed to identify the apoptotic role of ASPP2 overexpression in the progression of NAFLD. We detected cell death by using the M30 antibody, which can specifically recognize the k18 fragments produced by caspase 3 cleavage during the apoptotic process. In our HepG2 experiments, cell death was significantly lower after the OA stimulus and pre-incubation with ASPP2-ad compared with after the OA stimulus and pre-incubation with GFP-Ad alone (Fig. 2A). Upon counting the M30-positive cells (200 cells/well in three random wells), we found that the number of cells stained positive for M30 (8.8 ± 3.3%) in the OA plus GFP-Ad-treated group was higher compared with the OA plus ASPP2-Ad group (3.7 ± 1.5%; Fig. 2B).

We then measured the levels of LDH released from the OA plus GFP-Ad and OA plus ASPP2-Ad groups (*i.e*., the levels released into the culture medium) to identify necrosis and late apoptosis in the differently treated HepG2 cells. The levels of LDH in the culture medium did not significantly differ between the OA plus GFP-Ad and OA plus ASPP2-Ad-treated HepG2 cells (Fig. 2C). We then confirmed this finding by examining the ultrastructure of the HepG2 cells by using TEM (Fig. 2D); the numbers of apoptotic bodies in the OA plus ASPP2-Ad-treated HepG2 cells were significantly lower compared with the OA plus GFP-Ad-treated HepG2 cells. Therefore, ASPP2 might protect HepG2 cells from OA-induced early apoptosis.

### Reduced autophagy by ASPP2 contributed to OA-treated HepG2 survival

**Fig. 3:**
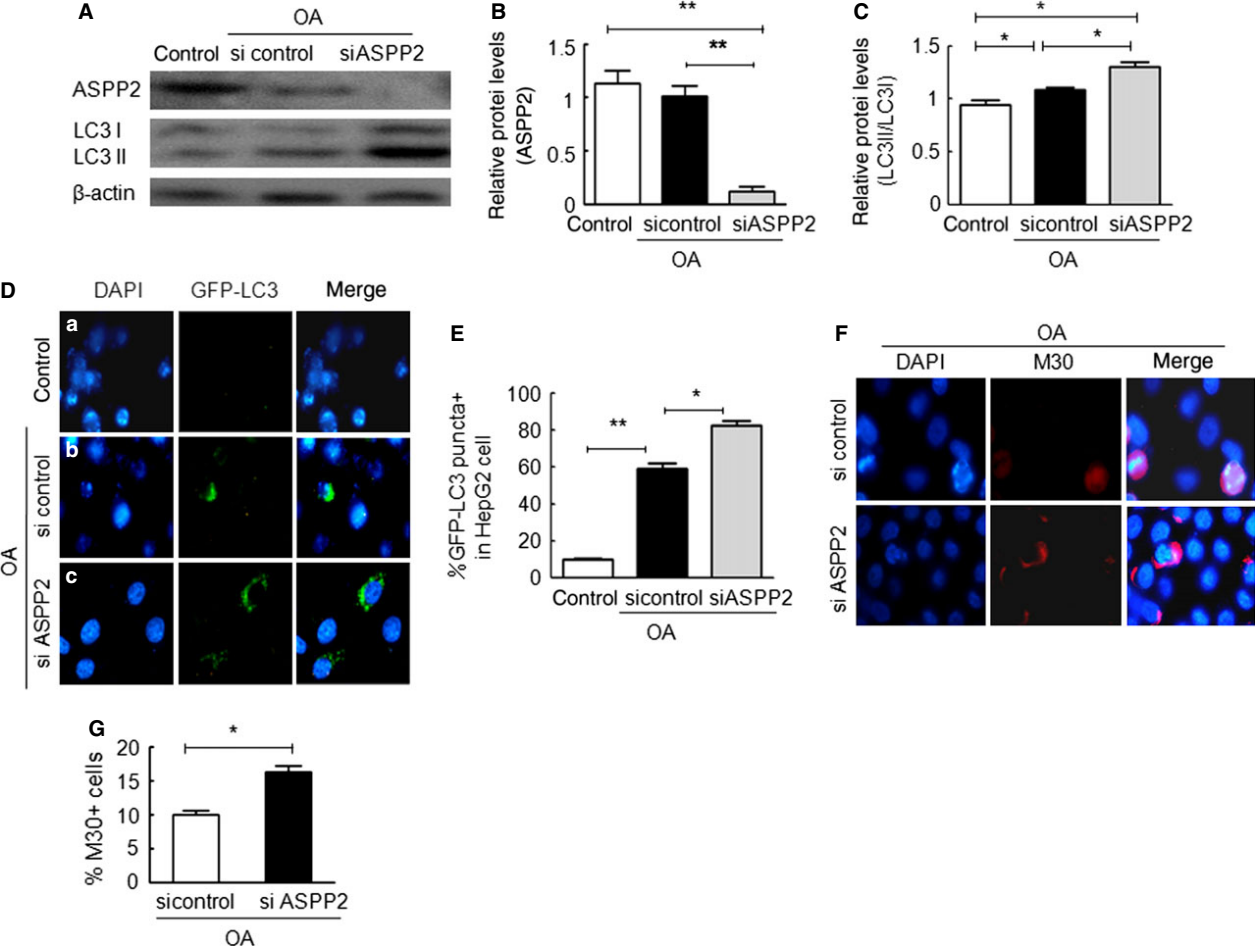
ASPP2 siRNA promoted autophagy and apoptosis. (**A**) The detection of autophagy using western blotting. (**B** and **C**) The relative protein levels of ASPP2 and LC3II/LC3I were normalized with β-actin. (**D** and **E**) The detection of autophagy by using immunofluorescence observation. (**F** and **G**) The detection of apoptosis using anti-M30. Original magnification (400×); *P < 0.05; **P < 0.01.

In the results described earlier, ASPP2 overexpression reduced the autophagy and apoptosis of OA-treated HepG2 cells. We wondered whether the autophagy reduced by ASPP2 was associated with the death of the OA-treated HepG2 cells. First, we detected the level of ASPP2 expression by using WB analysis of an untreated control, an OA plus ASPP2 siRNA control, and an ASPP2 siRNA control in HepG2 cells. ASPP2 expression was induced by OA treatment and knocked down by ASPP2 siRNA (Fig. 3A upper row and Fig. 3B). The ratio of LC3-II/LC3-I was 1.2-fold higher in the OA plus ASPP2 siRNA treatment group compared with OA alone, which was statistically significant (Fig. 3A middle row and Fig. 3C). Immunofluorescence examination of GFP-LC3 puncta and ASPP2 expression also confirmed that the number of GFP-LC3 puncta was significantly higher in the OA plus ASPP2 siRNA-treated HepG2 cells (Fig. 3D-c *versus* -b and Fig. 3E). In a cell apoptosis assay based on M30 immunofluorescence staining, the percentage of M30-positive cells in the OA plus control siRNA-treated HepG2 cells (10 ± 1%) was lower compared with the OA plus ASPP2 siRNA-treated HepG2 cells (16.3 ± 1.5%; Fig. 3F and G). These results further confirmed that ASPP2 can significantly reduce the level of autophagy and has a protective role in OA-treated HepG2 cells.

### The ASPP2-mediated inhibition of autophagy contributes to the TG reduction in a mouse NASH model

**Fig. 4:**
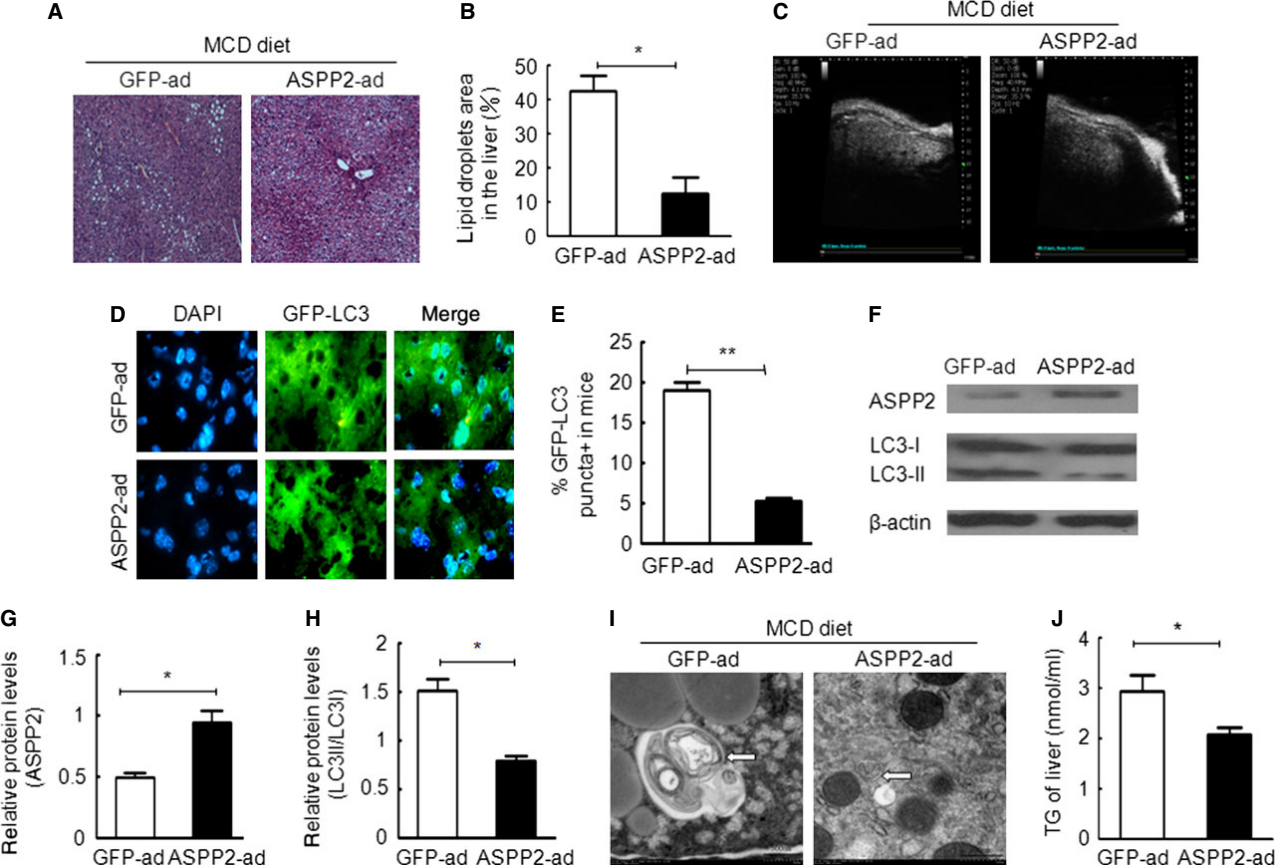
ASPP2 decreased the triglyceride content and inhibited autophagy *in vivo*. ASPP2++ mice were injected with GFP-ad as the control. ASPP2++ mice were injected with ASPP2-ad as the experimental group (ASPP2-ad group). All mice were fed a MCD diet for 10 days. (**A** and **B**) Haematoxylin-eosin staining revealed markedly diminished micro- and macrovesicular steatosis in the livers of the ASPP2-ad injected mice. Representative images are shown (100×). (**C**) Ultrasonic examination. (**D** and **E**) The detection of autophagy was performed with immunofluorescence. (**F**) The detection of autophagy by using western blotting analysis. (**G** and **H**) Relative protein levels of ASPP2 and LC3II/LC3I were normalized and using β-actin. (**I**) An electron microscope image illustrating the morphology of autophagic vesicles. (**J**) The TG content in the liver; *P < 0.05; **P < 0.01.

Our study with OA-treated HepG2 cells showed that ASPP2 decreased the level of TGs and inhibited autophagy. We further investigated this result in a mouse model. The NAFLD model of BALB/c mice is described in the supplementary data. Murine livers experience mild to moderate steatosis after 10 days on a MCD diet, and the lipid droplets in the hepatocytes presented as a mixture of large and small bubbles (Fig. 4A and B). In a clinical setting, small-bubble lipid drops are reversible in fatty liver patients. Therefore, the mice fed a MCD diet for 10 days were used to determine the effect of ASPP2 on the TG levels and autophagy.

The GFP-LC3 expression vector and ASPP2-Ad were injected into the MCD mice *via* the tail vein. Forty-eight hours later, liver ultrasounds indicated echo enhancement in the GFP-ad group, which showed light to moderate fatty degeneration. Interestingly, the liver echo was weaker in the ASPP2 group compared with the GFP-ad group (Fig. 4C). Immunofluorescence staining and WB analysis showed that the number of GFP-LC3 puncta and the LC3-II/LC3-I ratio in the ASPP2-Ad group were significantly lower compared with the GFP-ad group (Fig. 4D–H). The EM data also indicated that ASPP2 overexpression inhibited autophagy. The autophagic vesicles were much larger and contained several varieties of organelles in the GFP-ad group compared with the ASPP2-ad group (Fig. 4I).

Overall, we found that ASPP2 inhibited autophagy *in vivo*. Next, we determined whether ASPP2 overexpression affected the *in vivo* TG levels. The TG levels in the liver tissue were significantly lower in the ASPP2-ad group compared with the control group (Fig. 4J). The liver lipid droplet areas were 10% and 40% of the total area in the ASPP2-ad group and GFP-ad group, respectively (Fig. 4B), which is consistent with the autophagy occurrence rates of 7% in the ASPP2-ad group and 18% in the GFP-ad group (Fig. 4E). Taken together, these data suggest that ASPP2 overexpression may reduce the TG levels by inhibiting autophagy.

### ASPP2 overexpression contributes to cell survival by reducing liver failure and inhibiting apoptosis

**Fig. 5:**
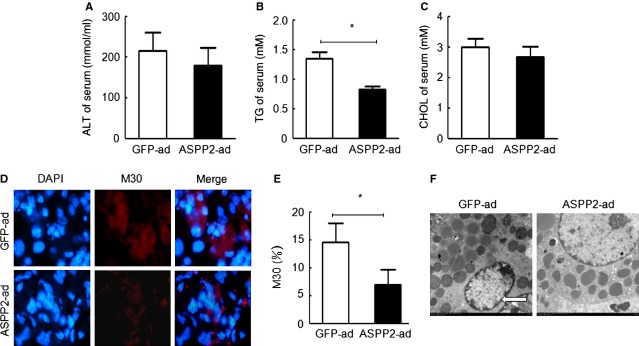
ASPP2 protects the liver from injury by inhibiting apoptosis. (**A**–**C**) The serum ALT levels, TG content, and CHOL content. (**D** and **E**) The detection of apoptosis by using an anti-M30 antibody. Original magnification (400×). (**F**) The detection of apoptosis in electron micrographs; *P < 0.05; **P < 0.01.

We demonstrated that ASPP2 overexpression reduced the level of TG by inhibiting autophagy and apoptosis *in vitro* and *in vivo*. Some papers have reported that autophagy affects the level of TGs in alcoholic fatty livers [25]. The level of TGs has also been shown to impact hepatic function. Furthermore, it is unknown whether liver function is influenced by autophagy and apoptosis in ASPP2-induced NAFLD. We further assessed the effects of ASPP2 overexpression on liver function in NAFLD mice that were fed a MCD diet. The serum ALT and CHOL were lower in the ASPP2-ad group compared with the GFP-ad group (Fig. 5A and C), while the TG content was lower not only in the serum but also in the liver of the ASPP2-ad group compared with the GFP-ad group (Figs. 4J and 5B).

Because hepatocytes may have been killed during MCD administration, M30 was used to detect early apoptosis [26–28]. Interestingly, the incidences of early apoptosis were 6.6 ± 4.5% and 14.5 ± 7.6% in the ASPP2-ad and GFP-ad groups, respectively (Fig. 5D and E). Small apoptotic bodies were observed under EM in the GFP-ad group, but not in the ASPP2-ad group (Fig. 5F). Taken together, these data suggest that ASPP2 overexpression may contribute to cell survival by the alleviation of liver failure and the inhibition of apoptosis.

### Decreased ASPP2 expression in NAFLD

**Fig. 6:**
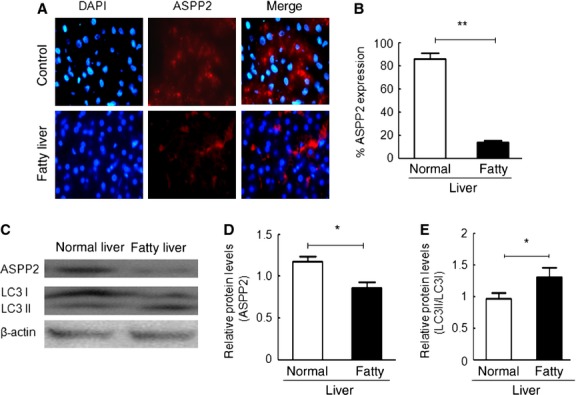
ASPP2 expression was decreased in the NAFLD liver. (**A** and **B**) Immunofluorescence images showing ASPP2 expression in the control and fatty liver. (**C**) The ASPP2 and LC3 levels detected by using western blot analysis. (**D** and **E**) The relative protein levels of ASPP2 and LC3II/LC3I were normalized by using β-actin; *P < 0.05; **P < 0.01.

Our findings indicate that ASPP2 overexpression attenuates the levels of TGs in the HepG2 cells treated with OA and in the BALB/c mice fed a MCD diet. Thus, we investigated ASPP2 expression in the clinical specimens of fat liver tissue from fatty liver patients. It is important to elucidate the role of ASPP2 in NAFLD. We observed the expression of ASPP2 in normal liver tissue and in liver tissue obtained from fatty liver patients. Immunofluorescence showed that ASPP2 expression was reduced in the fatty liver tissue compared with the control tissue (Fig. 6A and B). Western blot data demonstrated that ASPP2 is lower and the LC3-II/I ratio is higher in the NAFLD samples compared with the normal liver tissue (Fig. 6C–E), which further suggests that ASPP2 may play a role in NAFLD. The mechanism for this role requires further study.

## Discussion

With the growing incidence of obesity, sedentary lifestyles, and unhealthy dietary patterns, NAFLD has become a major health burden. At present, no effective method is available for comprehensively treating NAFLD. Continuous cell apoptosis is an indicator of NAFLD [23].

Recent studies have indicated that simple steatosis in NAFLD is reversible [24]. Autophagy changes play a role in the pathogenesis of NAFLD, but the relationship between autophagy and NAFLD remains controversial. Yuka indicated that NAFLD is associated with autophagic dysfunction. Hepatic steatosis has been found to suppress autophagic proteolysis [25]. However, studies by Ma demonstrated that mice with hepatocyte-specific autophagy deficiency were protected from fat accumulation in the liver (induced by a high-fat diet); these authors also observed decreased expression of specific genes involved in lipid metabolism [29]. Our finding is consistent Ma's results.

ASPP2 can regulate apoptosis in both p53-dependent and -independent pathways [30]. p53 can regulate autophagic activity by inducing LC3 expression [31]. N-terminal ASPP2, a known activator of p53, shares structural similarities with LC3 [32]. N-terminal ASPP2 inhibits autophagy. It is unknown whether ASPP2 regulates the level of autophagy through p53-dependent or -independent pathways.

To understand the functional role of ASPP2 in the regulation of an OA/MCD-induced model of NAFLD, we investigated whether ASPP2 overexpression could influence the TG content and cell survival by regulating autophagy. We found that ASPP2 overexpression contributed to cell survival, which was mediated by reducing the TG content and level of autophagy in cell and murine NAFLD models. Intracellular lipids are reportedly degraded in lysosomes when hepatocytes undergo autophagy [33]. In general, autophagy has been considered to play a protective role [34], but recent studies have reported that autophagy can play a dual role in cell protection and cell toxicity [35]. Ost reported the overactive autophagy was associated with large numbers of cytosolic lipid droplets [36]. Hagiwara demonstrated hyperactive autophagy under pathological conditions, including liver inflammation in a rat model of diabetes mellitus [37]. Komatsu described extensive liver autophagy during the catabolization of cytoplasmic components to free fatty acids [38], and diets rich in fatty acids promote fatty liver development [39], which indicates that excessive autophagy might induce fatty liver development. Our findings indicate that ASPP2 contributes to TG reduction by inhibiting autophagy. ASPP2 might reduce the level of TGs through its inhibition of hyperactive and toxic autophagy, which is consistent with the finding that hepatic steatosis is not exacerbated by hepatocyte-specific autophagy deficiency. Instead, autophagy blockade might accelerate the progression of non-alcoholic steatohepatitis. No inflammation occurs in the early stage of NAFLD.

Fatty degeneration is sometimes reversible during the early stages of NAFLD. NAFLD is characterized by the accumulation of lipids. Schleicher developed a mathematical model for the fate of fatty acids that are stimulated by hepatic lipid dynamics in hepatocytes. The model demonstrates reversion of the steatotic state back to the healthy state after reductions in the fatty acid uptake to below the threshold at which steatosis started [40]. Our findings demonstrated that fatty degeneration resulted in the appearance of a mixture of large and small bubbles in murine livers within 10 days of the MCD diet; the fatty degeneration of a small-bubble mixture can be reversed in the clinical setting. These data were obtained 10 days into our MCD mouse model and may represent the threshold for reversing steatosis. The ASPP2 overexpression group (with pre-incubation of ASPP2-ad by tail vein injection) had reduction in the fat or liver injury after 10 days. These findings suggest that ASPP2 plays a critical role in lipid metabolism, but the mechanism requires further study.

ASPP2 proteins interact with the DNA-binding domain of p53 through their SH3 and ankyrin domains. ASPP2 proteins promote the apoptotic function of p53; however, Bcl-2 anti-apoptotic proteins can combine with Ank-SH3 amino acids (the structural region of ASPP2 that is mainly responsible for regulating apoptosis) [41]. Recent studies have shown that Ddx42p-bound ASPP2 loses its apoptosis-stimulating activity in a manner that may be similar to the anti-apoptotic effect of Bcl-2 binding to Bbp [42]. Ddx42p is a DEAD protein related to Ddx17 (isoforms p72 and p82) [43]. Lipopolysaccharide is not only induced by p82 expression in mice [44], it is also associated with alterations in lipid content [45]. We found that ASPP2 acts as an anti-apoptotic agent *in vitro* and in the NAFLD mouse model. Further study is required to determine whether the excessive lipid accumulation observed in our NASH mouse model is reduced by the interaction between ASPP2 and Ddx42p. Interestingly, we demonstrated that ASPP2 inhibits autophagy and contributes to the inhibition of apoptosis, which is consistent with the finding that hyperactive autophagy is a possible cause of hepatocyte death in anorexia nervosa patients [46]. Taken together, these findings suggest that ASPP2 overexpression might represent a novel strategy for hepatocyte survival by decreasing the level of TGs in the treatment of NAFLD.

## Supporting information

**Figure S1** The construction of a fatty liver model of BALB/c mice.

**Figure S2** The construction of a fatty liver model of BALB/c mice.

**Figure S3** The autophagic levels in mice.

**Data S1** The construction of a fatty liver model in BALB/c mice.
