# Supplementary figures and images for: ASPP2 attenuates triglycerides to protect against hepatocyte injury by reducing autophagy in a cell and mouse model of non-alcoholic fatty liver disease
Source: J Cell Mol Med. 2014 Sep 25;19(1):155–64. doi: 10.1111/jcmm.12364 (PMC4288359; doi:10.1111/jcmm.12364)

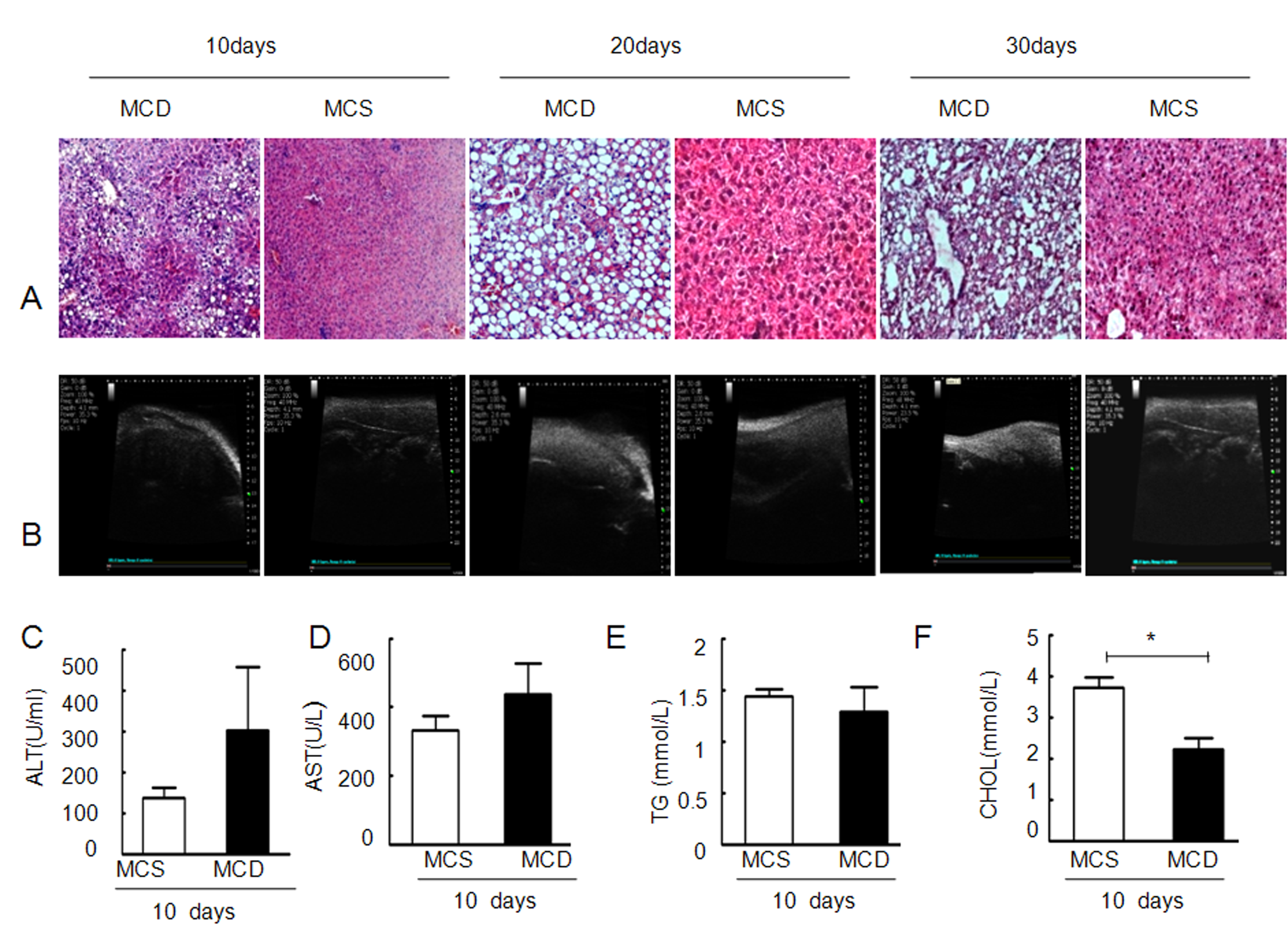

Supplement: Supplementary file 1 — Figure S1 The construction of a fatty liver model of BALB/c mice. [file jcmm0019-0155-sd1.tif]

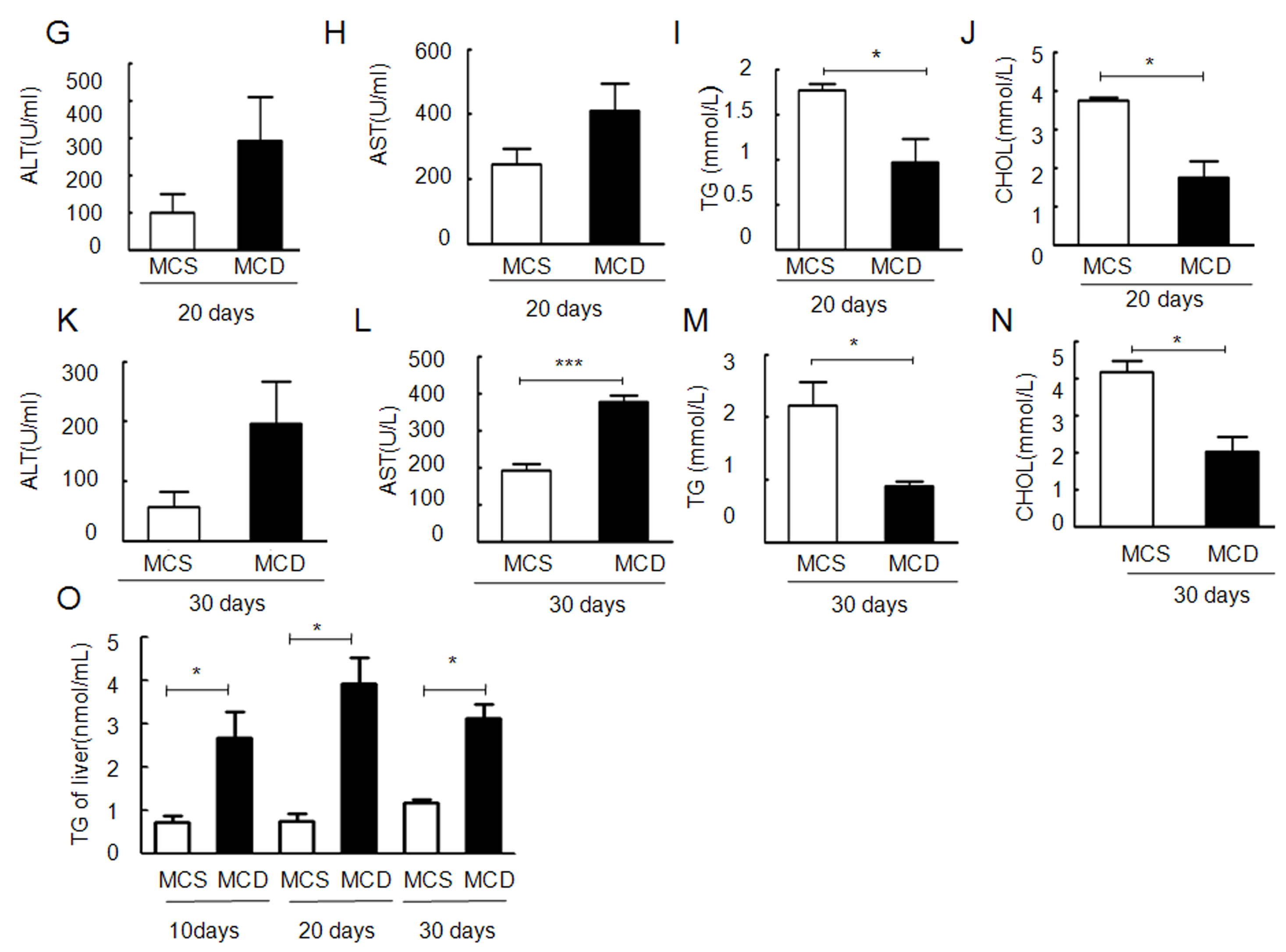

Supplement: Supplementary file 2 — Figure S2 The construction of a fatty liver model of BALB/c mice. [file jcmm0019-0155-sd2.tif]

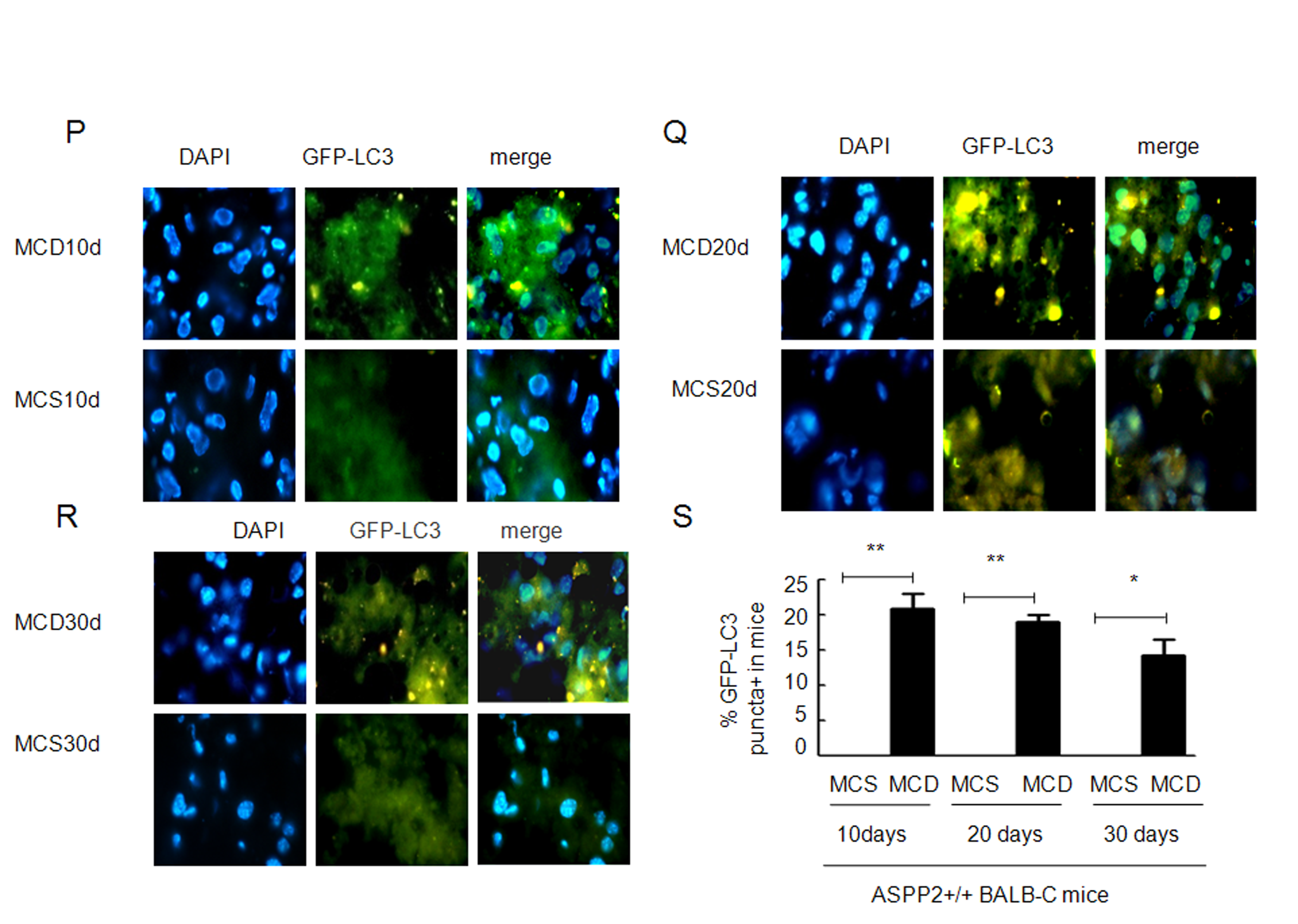

Supplement: Supplementary file 3 — Figure S3 The autophagic levels in mice. [file jcmm0019-0155-sd3.tif]
