# Supplementary material for: ASPP2 attenuates triglycerides to protect against hepatocyte injury by reducing autophagy in a cell and mouse model of non-alcoholic fatty liver disease
Source: J Cell Mol Med. 2014 Sep 25;19(1):155–64. doi: 10.1111/jcmm.12364 (PMC4288359; doi:10.1111/jcmm.12364)
Supplement: Supplementary file 4 — Data S1 The construction of a fatty liver model in BALB/c mice. [file jcmm0019-0155-sd4.doc]

*Supplementary data*

The construction of a fatty liver model in BALB/c mice

To determine whether the triglyceride (TG) levels decreased in response to treatment with ASPP2-ad over-expression in vivo, BALB/c mice were fed a methionine-choline-deficient (MCD) diet for 10, 20, or 30 days, which generated a fatty liver model for studying the relationship between TGs and autophagy. A methionine-choline-supplemented (MCS) diet was used in the control group.

Liver injury and steatosis were assessed by histopathological analyses. The BALB/c mice fed a MCD diet for 10 days had an accumulation of intracellular lipids in the liver, which was accompanied by reductions in the levels of circulating TG and cholesterol in the serum and was characterized as mild to moderate fatty liver disease. The lipid droplets appeared to be a combination of large and small bubbles that originated in the hepatic portal vein due to a poor blood supply (Fig. S1- A).The BALB/c mice that were fed a MCD diet for 20 and 30 days were characterized by moderate and severe fatty liver disease, respectively, based on pathologic slices （Fig.S1-A). However, the mice in the control groups that were fed MCS diets for 10, 20, and 30 days had no fatty steatosis (Fig. S1-A). To confirm that the construction of the nonalcoholic fatty liver disease mouse model in the BALB/c mice was successful, we used ultrasound technology and measured the ALT, AST, and CHO levels in the mouse serum. Based on the ultrasound measurements, the liver echoes slightly increased in the mice that were fed a 10-day MCD diet (Fig. S1-B); the liver echoes indicated moderate steatosis and liver disease. The angle was dull in the mice fed a 20-day MCD diet (Fig.S1-B), which suggests that the liver contained a small volume of water. The liver echo significantly increased in the mice that were fed a 30-day MCD diet, which indicated severe steatosis (Fig.S1-B). The mice that were fed the MCS control diet had no increase in their liver echoes (Fig.S1-B).

We assessed the nonalcoholic fatty liver disease model in BALB/c mice by measuring the ALT, AST, TG, and CHO levels. The ALT and AST levels in the MCD diet mice were higher compared with the MCS diet mice (Fig. S1 C-F，Fig. S2 G-N). The AST content in the MCD diet mice was significantly higher compared with that in the mice fed the 30-day MCS diet （Fig. S2 L）, which indicated the presence of liver necrosis. As time progressed, the TG and CHO levels in the serum of the MCD diet mice gradually decreased compared with the MCS group (Fig. S1E- F, Fig. S2 I-G, M-N), and the TG levels in the livers of the MCD diet mice gradually increased compared with the MCS group, which demonstrated that TGs accumulated in the liver (Fig. S2 O).

Autophagy levels increased in the BALB/c MCD mouse model

Because autophagy levels were reportedly increased in a NASH mouse model, we determined the autophagy levels in the BALB/c MCD mouse model. The autophagy levels significantly increased in the mice that were fed the MCD diet compared with the mice fed the MCS diet for 10, 20, and 30 days (Fig. S3 P-S). The autophagy levels reached their highest levels at 10 days and then gradually decreased at 20 and 30 days in the MCD diet mice (Fig. S3 P-S). In conclusion, we succeeded in building a model of nonalcoholic fatty liver disease in BALB/c mice.
